# Exploring the effect of different tea varieties on the quality of Sichuan Congou black tea based on metabolomic analysis and sensory science

**DOI:** 10.3389/fnut.2025.1587413

**Published:** 2025-05-09

**Authors:** Liran Yang, Xueping Luo, Qi Wang, Mingli Liu, Jingna Yan, Congming Wang, Yuanhua Xian, Kangli Peng, Kunyi Liu, Bin Jiang

**Affiliations:** ^1^School of Modern Agriculture & School of Wuliangye Technology and Food Engineering, Yibin Vocational and Technical College, Yibin, China; ^2^Research Institute of Tea Industry of Yibin, Yibin, Sichuan, China

**Keywords:** tea varieties, Sichuan Congou black tea, metabolomics, taste, correlation analysis

## Abstract

Sichuan Congou black tea (SCGBT), one of China’s top three high-aroma black teas, enjoys widespread consumer popularity. However, research into optimal processing varieties remains insufficient. This study assessed the quality of SCGBT produced from eight tea varieties: ‘*Fudingdabai*’ (FDDB), ‘*Zhongcha 302*’ (ZC302), ‘*Wuniuzao*’ (WNZ), ‘*Chuancha* 2’ (CC2), ‘*Fuxuan* 9’ (FX9), ‘*Mingshan 131*’ (MS131), ‘*Zhongcha 108*’ (ZC108), and ‘*Huangjinya*’ (HJY). Sensory evaluation, quantitative chemical analysis, and metabolomics techniques were employed. Results indicated that HJY, ZC302, and MS131 exhibited strong sweetness, umami, and mellowness, while CC2, FX9, and ZC108 were characterized by intense bitterness and astringency, attributed to high tea polyphenol levels and low free amino acid concentrations. The quality of WNZ and FDDB was relatively average. Using non-targeted metabolomics, 4,476 metabolite ion features were detected, and 75 significantly differential metabolites were identified (*p* < 0.05 and VIP > 1.0). Correlation analysis revealed that metabolites such as valine, L-glutamic acid, asparagine, L-serine, kaempferol-3-O-sophoroside-7-O-glucoside, quercetin-7-O-glucoside, L-tyrosine, and L-norleucine significantly influenced the taste of the tea infusion (|*r*| > 0.8, *p* < 0.05). Elevated levels of amino acids (L-glutamic acid and asparagine) and phenolic acids (gallic acid) contributed to the umami of HJY, ZC302, and MS131. Astringency was primarily determined by variations in flavonol/flavone and flavonol/flavone glycosides, as well as ester catechins. Additionally, the content of sweet-tasting amino acids and non-ester catechins was found to fluctuate. In summary, ZC302, MS131, and HJY are more suitable for processing into higher-grade SCGBT. These findings provide a theoretical and practical foundation for selecting and breeding high-quality SCGBT varieties and ensuring consistent product quality.

## 1 Introduction

Black tea, one of the six major tea categories in China, is a fully fermented tea known for its sophisticated processing methods, rich historical and cultural heritage, health benefits, and distinctive high aroma and flavor. Representing approximately 75% of global tea sales (1–3), black tea is produced from a variety of tea plant cultivars across different countries and regions, each lending unique flavor profiles to the final product (1, 4, 5). For instance, Dianhong, made from the fresh leaves of the Yunnan large-leaf variety, is characterized by a caramel-like aroma and a “mellow” flavor (6, 7). Keemun black tea, processed from the small-to-medium-leaf Zhuyezhong variety, is famous for its floral and fruity fragrance, often referred to as the “Keemun aroma,” and its sweet, mellow taste (8, 9). Xinyang black tea, derived from the Xinyang cultivar, boasts a honey-like aroma primarily attributed to phenylacetaldehyde (10). Assam black tea from India is known for its malty and rose-like scent (11), while Ceylon black tea from Sri Lanka is distinguished by its floral notes and lingering sweet aftertaste (12).

Sichuan, a historically significant tea-producing region in China, is a major source of black tea, with Sichuan Congou black tea (SCGBT) being a notable variety. SCGBT, renowned for its tangerine aroma and sweet taste, is produced through withering, rolling, fermentation, drying, and refining processes. As one of China’s three most famous high-aroma black teas, alongside Keemun and Dianhong, SCGBT accounts for about 10% of the national black tea production and is highly favored by consumers (13). In recent years, the tangerine aroma of SCGBT has attracted considerable research attention. Studies utilizing gas chromatography and gas chromatography–mass spectrometry have identified numerous volatile compounds, revealing key aroma substances responsible for its distinctive tangerine scent (2, 13). Additionally, metabolomics techniques have been employed to explore taste component variations among SCGBTs of different grades and origins (14, 15). However, most prior research has primarily focused on the volatile and non-volatile substance changes during SCGBT processing (2, 13, 16), with limited investigation into the sensory quality and taste component differences among various SCGBT cultivars. The variety of tea plants significantly influences the physical properties, taste, and aroma of the fresh leaves, which in turn impacts the quality of the final tea product (17–19). Consequently, selecting appropriate tea varieties is essential for producing high-quality SCGBT, highlighting the need for further in-depth research into suitable cultivars.

The characteristic metabolites in black tea, including tea polyphenols (TPs), catechins, caffeine (CAF), amino acids, and theaflavins, not only contribute to the aroma, infusion color, and taste of the tea, but also offer health benefits such as antioxidation, blood sugar reduction, blood pressure regulation, and modulation of intestinal microbiota, which are closely linked to human health (20–22). High-performance liquid chromatography (HPLC) and liquid chromatography-mass spectrometry (LC–MS) techniques are widely used for the separation, qualitative, and quantitative analysis of these active ingredients due to their high sensitivity, resolution, and effective chromatographic separation performance (1, 13, 23). These methods have led to the identification of secondary metabolites in black and white teas, such as theaflavin, theaflavin-3’-O-gallate, theaflavin-3-O-gallate, theaflavin-3,3’-O-digallate, and N-ethyl-2-pyrrolidinone-substituted flavan-3-ols, each exhibiting various bioactivities (24, 25). However, research on the optimal cultivars for SCGBT remains limited. ‘*Fudingdabai*’ (FDDB), ‘*Zhongcha 302*′ (ZC302), ‘*Wuniuzao*’ (WNZ), ‘*Chuancha 2*′ (CC2), ‘*Fuxuan 9*′ (FX9), ‘*Mingshan 131*′ (MS131), ‘*Zhongcha 108*′ (ZC108), and ‘*Huangjinya*’ (HJY) are among the most commonly used tea cultivars for black tea production in Sichuan, China, with large-scale cultivation (14, 26). This study, therefore, employed sensory evaluation and metabolomics techniques to assess the quality of SCGBT produced from these eight cultivars, aiming to investigate the impact of tea plant variety on SCGBT quality and provide valuable insights for selecting varieties suitable for SCGBT production and improving its overall quality.

## 2 Materials and methods

### 2.1 Chemical reagents

Ferrous sulfate, potassium sodium tartrate, disodium hydrogen phosphate, potassium dihydrogen phosphate, 95% ethanol, ninhydrin, aluminum chloride, sodium hydroxide, 2-Chloro-L-phenylalanine, and formic acid (AR grade) were obtained from China National Pharmaceutical Group and Shanghai Chemical Reagent Co., Ltd. Chromatographic-grade methanol and acetonitrile were purchased from Merck (Darmstadt, Germany). OPA reagent (10 mg/mL) and borate buffer (0.4 M, pH 10.4) for derivatization were sourced from Agilent Technologies (Palo Alto, CA, USA). Authentic standards were purchased from Chengdu Must Biotechnology Co., Ltd. (Chengdu, China), Sigma-Aldrich (St. Louis, MO, USA).

### 2.2 Manufacturing process and collection of tea samples

The raw materials for SCGBT processing were fresh tea leaves (*Camellia sinensis* cv. FDDB, ZC302, WNZ, CC2, FX9, MS131, ZC108, and HJY, one bud and one leaf) harvested on March 16, 2024, in Yibin County, Sichuan Province, China. The SCGBT was produced through traditional processing methods, including withering, rolling, fermentation, drying, fragrance enhancement, and refining, under the supervision of a tea master with over 10 years of experience at the Sichuan Congou Engineering and Technology Research Center, Yibin Vocational and Technical College. Briefly, 30 kg of fresh tea leaves from each variety were divided into three equal portions and withered in a wilting tank at an ambient temperature of 24–26°C and relative humidity of 70–75%. The leaf thickness was maintained at 2–3 cm, and the leaves were turned every 5 h until the water content was reduced to 62–64%, a process lasting approximately 22 h.

A barrel-type rolling machine (6CR–35, 35 cm diameter, Sunyoung Machinery Co., Ltd., Zhejiang, Quzhou, China) was then used to roll the withered leaves at 30 rpm for 1.5 h, comprising 25 min of light pressure, 30 min of medium pressure, 25 min of heavy pressure, and 10 min of light pressure. Following rolling, the leaves were fermented in a fermentation room (22–25°C, moisture content ≥ 95%) for 4.5 h. The fermented leaves were then dried at 110°C using a 6CTH-60 type drying machine for 15 min, followed by 1 h of piling, and then further dried at 80°C until sufficiently dry. Finally, a winnowing machine (EF-40, Sunyoung Machinery Co., Ltd., Zhejiang, Quzhou, China) was employed to remove impurities such as old leaves and stems. The processed black tea was then spread in a box-type hot air drying machine (JY-6CHZ-7B, Fujian Jiayou Machinery Co., Ltd., Fujian, China) and dried at 80°C for 3 h. The samples were stored at −20°C for further analysis.

### 2.3 Traditional sensory evaluation

SCGBT samples were evaluated by a panel of seven trained assessors (three males and four females, aged 27 to 40 years), all holding the national senior tea evaluator professional qualification certificate, in accordance with the national standard (GB/T 23776-2018). The samples were randomly encoded with three-digit numbers to mask their identity, and each tea sample was independently evaluated by the panelists. Each sample underwent three repeated evaluations. Each SCGBT sample (3.0 g) was brewed in boiling water (150 mL) for 5 min to obtain the infusion used for taste evaluation. The attributes assessed included infusion color, aroma, taste, and appearance of the infused leaves. The total score of overall sensory quality was calculated using the formula: Total score = appearance × 25% + infusion color × 10% + aroma × 25% + taste × 30% + brewed tea leaves × 10% (27). The sensory ranking of SCGBT samples was determined based on the comprehensive evaluation provided by the panelists.

### 2.4 Quantitative descriptive analysis of the taste

Following the traditional sensory evaluation, the taste characteristics of SCGBT produced from different tea varieties were assessed. All seven evaluators were specifically trained to identify, describe, and quantify various taste characteristics. Six key taste descriptors were selected, representing common taste qualities: sweetness, umami, bitterness, sourness, mellowness, sweet aftertaste, and astringency. Each attribute was evaluated on a 5-point scale: 5 for extremely strong intensity, 4 for strong, 3 for neutral, 2 for weak, 1 for very weak, and 0 for none (13, 28). The intensity of each sensory attribute was determined based on the average ratings from the seven panelists.

### 2.5 Analysis of primary quality components

Water extract (WE) and free amino acid (FAAs) contents were determined according to the guidelines of the National Standard of China GB/T 8305–2013 and GB/T 8314–2013, respectively. TPs were quantified using the Folin–Ciocalteu method with GA as a standard at a wavelength of 765 nm (GB/T 8313–2018). The contents of theaflavins (TFs), thearubigins (TRs), and theabrownins (TBs) in SCGBT were analyzed by a spectrophotometric method as described previously (29).

### 2.6 HPLC analysis of caffeine, catechins, flavones, and amino acids

For the determination of GA, CAF, catechins, and flavones, high-performance liquid chromatography (HPLC) was performed using a Shimadzu LC–16 system (Shimadzu, Japan) equipped with a photodiode array detector, as described in our previous study (13). Total ester catechins were calculated as the sum of EGCG, GCG, CG, and ECG, while total non-ester catechins were calculated by summing C, EC, GC, and EGC. The catechin bitterness index (IC) was calculated based on methods described by Jiao et al. (30) and Qiao et al. (31). The contents of FAAs in tea leaves were analyzed using the HPLC method described by Zhao et al. (32).

### 2.7 Non-targeted metabolomics analysis

The sample extraction method was modified based on previous methods (33, 34). Briefly, 0.05 g of tea powder and 1 mL of an acetonitrile–methanol–water (45:45:10, v/v/v) solution containing N-acetyl-D-alloisoleucine (20 mg/L) as an internal standard were added to a 5 mL centrifuge tube. The mixture was then subjected to ultrasonication for 10 min and left to stand for one hour at −20°C. Next, the supernatant was centrifuged at 12,000 g for 15 min at 4°C. The supernatant was then aspirated and subjected to liquid chromatography-mass spectrometry (LC–MS) analysis at Biomarker Technologies Co., Ltd. (Beijing, China). Triplicate preparations and analyses were performed for each sample. Detailed approaches are described in Supplementary material.

### 2.8 Statistical analysis

All analyses were performed with at least three biological replicates and expressed as the mean ± standard deviation (SD). The taste radar plot was created using Excel 2019 software (Microsoft Corporation, WA, USA). Bar charts and heatmaps were visualized using GraphPad Prism (version 9) and the integrative toolkit TBtools. Chord diagram were created using the website-based tool Chiplot.^1^ One-way analysis of variance (ANOVA) with least significant difference (LSD) and Pearson correlation analysis were performed using SPSS (version 22.0; Chicago, IL, USA). If the *p* value < 0.05, the comparisons were regarded as statistically significant. Principal component analysis (PCA), orthogonal partial least squares discriminant analysis (OPLS–DA), partial least squares discriminant analysis (PLS–DA), and hierarchical cluster analysis (HCA) were conducted using SIMCA-P 14.1 software (Umetrics AB, Umeå, Sweden). Significantly regulated metabolites between groups were determined by variable importance in projection (VIP) > 1 and *p* < 0.05. VIP values were extracted from the OPLS–DA results. Co-expression networks of compounds were created using the website-based tool Chiplot.^2^

## 3 Results and discussion

### 3.1 Effects of different tea varieties on the sensory quality of SCGBT

**Figure 1:**
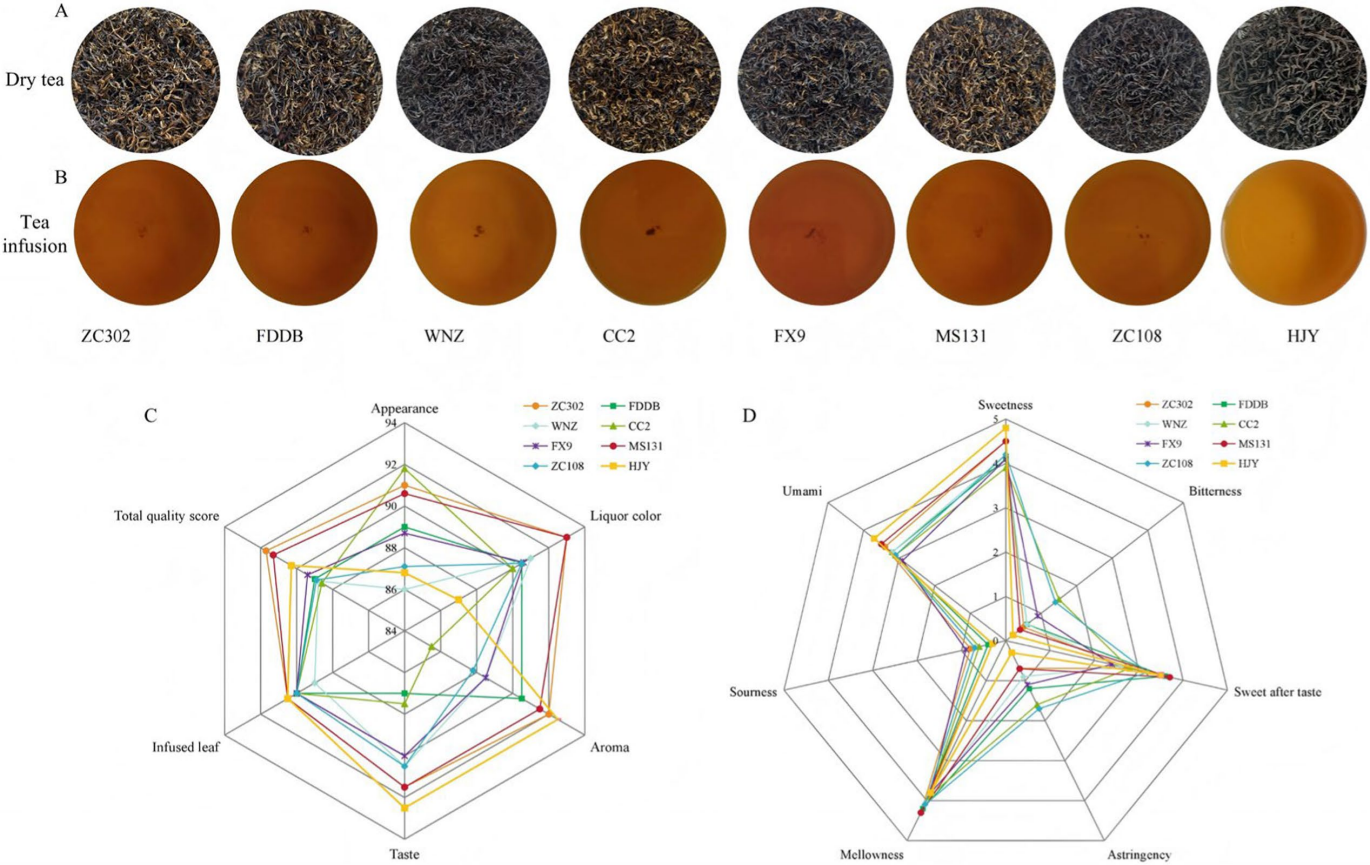
**(A)** Displays images of the tea’s appearance, infused leaves, **(B)** Tea infusion. **(C)** Sensory evaluation results of different varieties of SCGBT. **(D)** Quantitative descriptive analysis radar map of taste for different varieties.

The quality of tea is strongly influenced by the variety of the tea tree. In this study, sensory evaluations were conducted on eight SCGBT samples from different tea tree varieties to comprehensively analyze the quality differences. The results are presented in Supplementary Table S1 and Figures 1A–C. In terms of appearance, the most noticeable variation was the color of the dry tea. Specifically, ZC302, CC2, and MS131 displayed more golden pekoe than the other varieties, while WNZ, ZC108, and HJY showed fewer golden pekoe, and the other varieties had a moderate amount. The tea infusion colors were predominantly red and bright, with the exception of HJY, which exhibited an orange-red and bright color. Regarding aroma, HJY and ZC302 exhibited sweet and fruity aromas, while MS131 had a strong sweet and long-lasting floral scent. CC2 and ZC108, however, had slightly grassy aromas, and the other SCGBT samples had a predominantly sweet scent. The taste characteristics of the eight SCGBT samples varied significantly. HJY, ZC302, and MS131 had a mellow and thick taste with a sweet aftertaste. FDDB had slight sourness, while CC2 exhibited a slightly astringent taste, although sweetness remained the dominant flavor. Additionally, all SCGBT samples had tender, red, and bright color-infused leaves. Overall, these sensory results indicated that all selected teas had the typical characteristics of SCGBT, which could be used for subsequent research. There were, however, significant flavor differences between the different tea varieties of SCGBT, with HJY, ZC302, and MS131 having higher total quality scores than the other samples.

Quantitative descriptive analysis (QDA) is a widely used method for flavor evaluation, particularly for black tea (3, 10, 13). Figure 1D illustrates that the eight tea varieties of SCGBT exhibit significant differences in taste attributes. HJY stands out with distinct sweetness and umami attributes, which is likely due to its higher amino acid levels and lower tea polyphenol and catechin content (13, 35). ZC302 and MS131 had the highest mellowness attributes, while CC2 and ZC108 exhibited the highest bitterness and astringency. Other SCGBT samples often had sweetness attributes. In summary, the QDA results were consistent with traditional sensory evaluations, showing that the tea variety significantly impacts the sensory quality of SCGBT, affecting not only appearance and liquor color but also taste. To further investigate the taste components of SCGBT, the nonvolatile compounds in SCGBT were analyzed to explore how different varieties influence the taste quality.

### 3.2 Effects of different tea varieties on the contents of general biochemistry components of SCGBT

**Figure 2:**
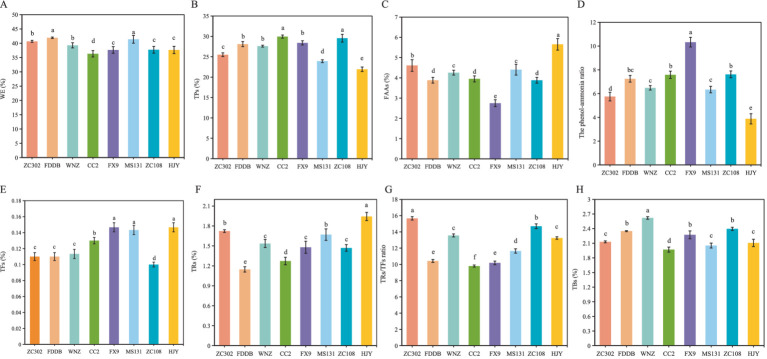
Differential primary quality components content of SCGBT processed by eight tea varieties. Panels **(A–H)** depict water extracts (WEs), tea polyphenols (TPs), free amino acids (FAAs), the phenol-ammonia ratio (TP/AA), theaflavins (TFs), thearubigins (TRs), the TRs/TFs ratio, and theabrownins (TBs). Content is expressed as the average value (%) ± standard deviation (*n* = 3), with different letters indicating significant differences at the 0.05 level.

WEs represent the soluble components in Gongou black tea, with their concentration directly influencing the tea’s strength and flavor. WEs primarily consist of TPs, soluble sugars, FAAs, CAF, and TFs (33, 36). As shown in Figure 2A, significant variations in WE content are observed in SCGBT processed from different tea tree varieties. Notably, MS131 and FDDB exhibit considerably higher WE levels than other varieties (*p* < 0.05), which likely accounts for the more pronounced mellow taste of SCGBT derived from these two cultivars. In contrast, no significant differences in WE content are noted among ZC302, WNZ, FX9, ZC108, and HJY, with CC2 having the lowest WE content. TPs, key contributors to tea bitterness and health-promoting properties (33, 37), Figure 2B show the highest levels in CC2 and ZC108 at 29.96 and 29.57%, respectively, both significantly higher than those in other varieties (*p* < 0.05). HJY, however, exhibits the lowest TPs content at 21.93%, which may be attributed to the specific genetic traits of this variety (13). FAAs, essential quality components, enhance the freshness and smoothness of black tea (17, 28). Figure 2C demonstrates a significant variation in the FAAs content across SCGBT produced from eight tea tree varieties. HJY exhibits the highest FAA content at 5.65%, which is linked to the yellowing of tender shoots and the high amino acid concentration inherent to the HJY cultivar (13). In contrast, FX9 displays the lowest FAA content at 2.75%, likely due to the relatively fewer golden pekoe present in the SCGBT from this variety. Tea trichomes, known for their high amino acid content, can reduce bitterness and astringency, enhance freshness, and improve floral and fruity aromas in white tea (38). In black tea, trichomes contribute to the brightness and freshness of the infusion (39). The phenol-ammonia ratio (TP/AA) serves as a critical indicator of the freshness and briskness of the tea infusion, as well as the suitability of specific tea varieties for black tea production. A lower TP/AA ratio is indicative of a softer, fresher, and more brisk infusion (17). Statistical analysis reveals that FX9, CC2 and ZC108 exhibit higher TP/AA ratios, while ZC302 and HJY show lower ratios (Figure 2D).

TFs are the primary contributors to the taste and infusion color of black tea, playing a pivotal role in the characteristic golden yellow color of high-quality infusions (40). Higher TFs content correlates with increased infusion brightness. Both HJY and MS131 exhibit significantly higher TFs levels compared to other varieties, which aligns with their superior sensory scores for soup color (Figure 2E). Thearubigins (TRs), the most abundant oxidation products in black tea, are primarily responsible for its red liquor color (Figure 2F). Research indicates that the TRs/TFs ratio in high-quality black tea should range from 10 to 15 (40). In this study, the TRs/TFs ratio for SCGBT from all varieties, except CC2, falls within this optimal range (Figure 2G). Theabrownins (TBs), which contribute to the dullness and lack of astringency in black tea infusion, are negatively correlated with tea quality. As shown in Figure 2H, WNZ contains significantly higher TBs levels than other varieties, while ZC302, MS131, and HJY have notably lower TBs content than the other varieties, except for CC2 (*p* < 0.05). These results suggest that SCGBT from ZC302, MS131, and HJY offer a fresher, mellower taste and a brighter yellow infusion, indicating higher quality than those from other tea varieties.

### 3.3 Effects of different tea varieties on the contents of catechins, flavones, and FAAs of SCGBT

**Figure 3:**
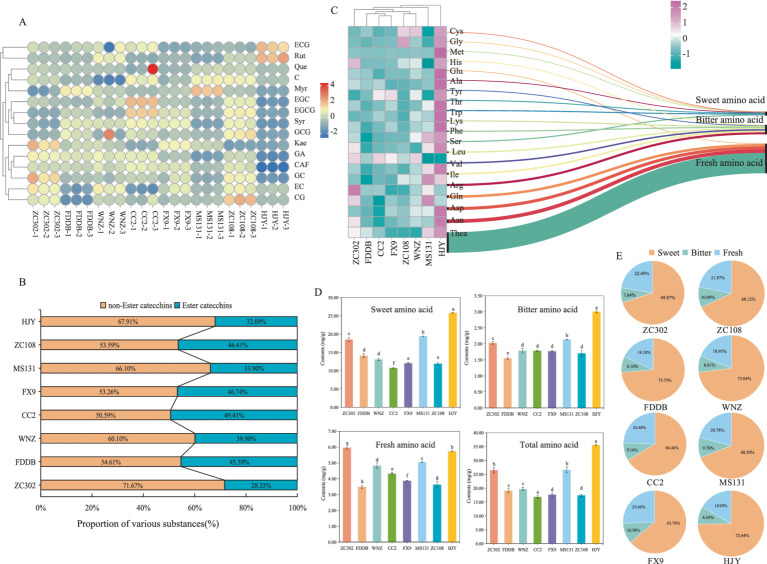
Comprehensive analysis of biochemical components of SCGBT. **(A)** Chord diagram of catechins and flavones content in different SCGBT varieties. **(B)** Composition proportion of catechins in eight SCGBT samples. **(C)** Heatmap of amino acid content in eight SCGBT samples. **(D)** Total amino acid content and the content of different flavor amino acids in eight SCGBT samples. **(E)** Composition proportion of amino acids in eight SCGBT samples.

Catechins, flavones, FAAs, and CAF are critical chemical components influencing tea flavor intensity (37). Catechins and CAF, in particular, contribute to the puckering astringency and bitterness of tea. A total of eight catechin monomers were identified in all tea samples (Figure 3A), including four ester catechins (CG, ECG, GCG, and EGCG) and four non-ester catechins (C, EC, GC, and EGC). Non-ester catechins impart a sweet aftertaste, while ester catechins produce a more astringent and stimulating effect (17, 41). As shown in Figure 3B, non-ester catechins were present in higher quantities than ester catechins. Among the eight SCGBT samples, the proportion and content of ester catechins were highest in CC2, with the ester catechin content following the order: CC2 > ZC108 > FX9 > FDDB > WNZ > MS131 > ZC302 > HJY. CC2 and ZC108 exhibited the highest total catechin content, with significantly higher levels of EGCG, EC, C, and ECG compared to the other varieties. Notably, EGCG, the predominant catechin in all samples, was present at significantly higher levels in CC2, FX9, and ZC108 compared to the other varieties (*p* < 0.05). EGCG is a major contributor to the bitterness and astringency of black tea liquor (41), while GCG and CG have been associated with astringency perception (42, 43). Both GCG and CG were higher in ZC108 than in the other varieties. Catechins accumulation is largely influenced by the genetic background of tea varieties, and variations in composition and content provide a theoretical foundation for selecting suitable varieties for black tea production.

GA is another compound contributing to the astringency of tea infusion (33, 41). Its content was relatively low in HJY and MS131 but higher in CC2, WNZ, and ZC302, with concentrations exceeding 5 mg/g in these varieties. Alkaloids in tea, including CAF, theanine, and theobromine, contribute significantly to tea’s bitterness, with CAF comprising more than 95% of the total alkaloid content (41, 43). In this study, HJY exhibited significantly lower CAF content compared to other varieties (*p* < 0.05), reaching only 43.8% of the highest level found in CC2. Flavones/flavonols and their glycosides, which impart a velvet-like astringency due to their low taste thresholds, are key metabolites in tea (41, 42). Of the five flavonols detected, Syr was the most abundant, with the highest content in CC2 and the lowest in HJY. Additionally, WNZ, CC2, and ZC108 contained higher total flavonol levels, while ZC302, MS131, and HJY had lower levels. These results suggest that ZC302, MS131, and HJY are more suitable for SCGBT production, as the SCGBT processed from these varieties exhibits lower bitterness compared to the other varieties.

The contribution of FAAs to tea flavor is primarily evident in their influence on freshness, sweetness, and bitterness, significantly enhancing the freshness and sweetness of black tea (13, 44). In this study, the contents of 20 amino acids were analyzed to better understand the quality differences across various SCGBT samples (Figure 3C). With the exception of Leu, Arg, and Gln, the concentrations of the remaining 17 amino acids in HJY were significantly higher than in the other seven varieties (*p* < 0.05). MS131 and ZC302 exhibited the highest levels of Arg and Gln. Furthermore, except for Tyr and Leu, the levels of 18 amino acids in FDDB, CC2, FX9, WNZ, and ZC108 were significantly lower compared to the other three varieties (*p* < 0.05). Theanine, a unique amino acid in tea, accounts for approximately 50% of the total amino acid content and is a key contributor to the freshness and briskness of black tea, along with glutamic acid (17, 31, 37). In this study, theanine comprised more than half of the total amino acids in all eight SCGBT samples, with HJY showing significantly higher levels of both theanine and glutamic acid compared to the other varieties (Figure 3C).

The 20 FAAs can be categorized based on their taste characteristics: fresh (Glu, Gln, Asp, Asn, Thea), sweet (Gly, Ala, Trp, Met, Cys, Thr, Ser), and bitter (Tyr, Arg, Phe, His, Lys, Ile, Leu, Val). The composition ratio of these taste-related amino acids was similar across the eight tea samples (Figure 3E), although there were differences in the absolute contents across varieties. Further analysis of the changes in amino acid content during processing (Figure 3D) revealed that the content of sweet, fresh, and total amino acids in ZC302, MS131, and HJY was significantly higher than in the other five SCGBT samples (*p* < 0.05). Conversely, the content of bitter amino acids followed an opposite trend, with ZC302, MS131, and HJY showing significantly higher levels than the other varieties (*p* < 0.05). The ranking of total amino acid content across the eight varieties was as follows: HJY > MS131 > ZC302 > WNZ > FDDB > FX9 > ZC108 > CC2. These results indicate that the amino acid content in SCGBT is heavily influenced by tea tree variety, with ZC302, MS131, and HJY having the potential to produce SCGBT with a fresher taste, consistent with sensory evaluation findings.

### 3.4 Screening for taste substances

**Figure 4:**
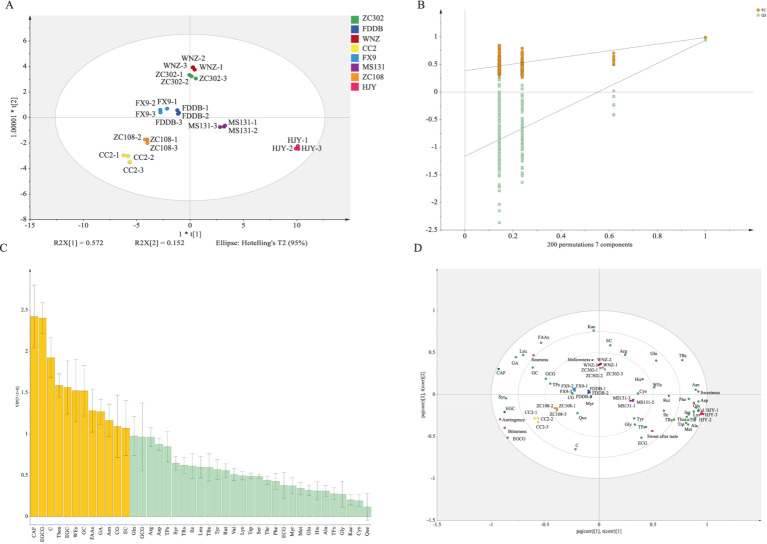
Multivariate statistical analysis of SCGBT samples from different varieties. **(A)** Score plot of OPLS–DA. **(B)** Permutation test (R^2^ = 0.386, Q2 = −1.163). **(C)** VIP plot. Yellow bars represent metabolites with VIP > 1; green bars represent metabolites with VIP < 1. **(D)** Loading plot.

OPLS–DA is a supervised multivariate statistical analysis method that filters orthogonal variables unrelated to classified variables, enhances group differences, and minimizes within-group variations, making it a powerful tool for screening differential metabolites (13, 37, 45). In this study, multivariate statistical analysis was conducted on all identified metabolites. The score plot reveals significant metabolic differences between the eight SCGBT samples, with good repeatability in the three parallel tests (Figure 4A, PC1 = 57.2%, PC2 = 15.2%), indicating pronounced metabolic variations. The 200 permutation tests for cross-validation show that the OPLS–DA discriminant model is not overfitted, confirming the model’s reliability (Figure 4B). Differential metabolites were identified based on the OPLS–DA model using VIP. Metabolites with VIP > 1 were considered to have significant contributions to the model (45). A total of twelve differential metabolites with VIP > 1 were selected, including CAF, EGCG, C, Thea, EGC, WEs, GC, FAAs, GA, Asn, CG, and EC (Figure 4C). These metabolites are primarily associated with sweetness, bitterness, freshness, and astringency, likely contributing to the taste differences among the eight SCGBT varieties. Additionally, Gln, GCG, and Arg exhibited VIP values near 1, suggesting their potential role in taste determination (Figure 4C). These pivotal differential metabolites were further highlighted in the loading plot to explore their specific contributions to quality differences between tea varieties (Figure 4D). The proximity of a metabolite to the cluster indicates its greater sensitivity to classification (46). Thea and Asn, which impart an umami taste, were closer to the HJY sample, while EC, contributing to a sweet aftertaste, was positioned near WNZ (41). Additionally, EGC and EGCG, associated with a strong bitter and astringent flavor and predominantly found in green and semi-fermented teas (41), were located near the CC2 and ZC108 samples.

### 3.5 LC–MS-based metabolomics analysis

**Figure 5:**
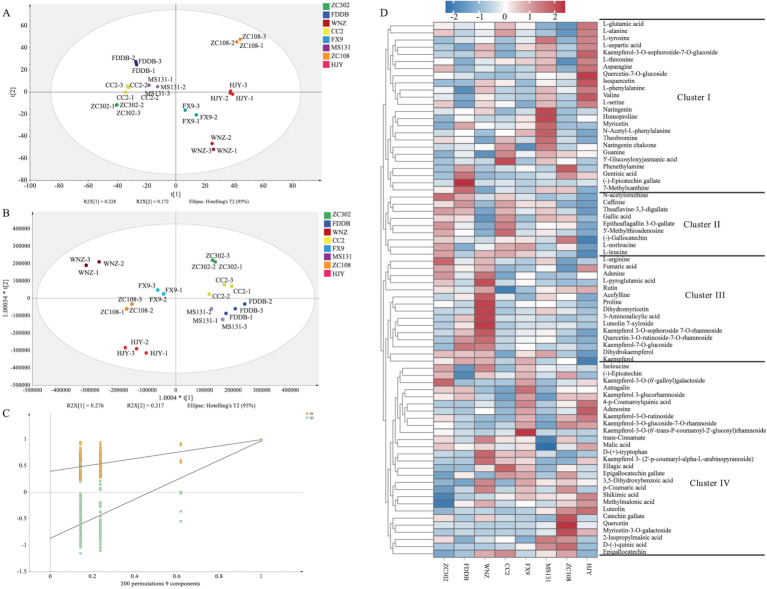
Multivariate statistical analysis of the metabolite profiles in SCGBT determined by LC–MS. **(A)** PCA score plots. **(B)** PLS-DA score plots (R^2^X = 0.946, R^2^Y = 0.992, Q^2^ = 0.891). **(C)** Permutation test (R^2^ = 0.397, Q^2^ = −0.869). **(D)** Heatmap of the 75 key metabolites in SCGBT samples from different tea varieties.

Although the main chemical components can account for the differences observed among SCGBT varieties, several flavor components influencing the quality of SCGBT remain undetected. To further investigate the taste differences, non-targeted metabolomics based on LC–MS was employed to identify taste-related substances. The high analytical precision of the presented metabolomics analysis is also supported by an R^2^ value of 0.9 between two parallel extractions of the same tea sample (Supplementary Figure S1). In total, 4,476 metabolite ion features were detected across the various SCGBT tea varieties based on exact mass measurement (< 5 ppm), MS/MS fragments, and retention time. All identified metabolites were subsequently used for multivariate statistical analysis. To elucidate the metabolic patterns of SCGBT processed from eight tea varieties, multivariate statistical analysis was performed. The unsupervised PCA model score plot (R^2^X = 0.86) showed clear clustering and differentiation of SCGBT samples from different tea varieties (Figure 5A). The first two principal components explained 22.8 and 17.2% of the variance, respectively, indicating significant differences in chemical quality between SCGBTs processed from different varieties. Following this, supervised PLS–DA was applied to further explore the metabolic differences between SCGBT samples (R^2^X = 0.946, R^2^Y = 0.992, Q^2^ = 0.891). The PLS–DA model effectively discriminated the SCGBT samples, which clustered into distinct regions, indicating pronounced metabolic differences among them (Figure 5B). The robustness of the model was confirmed through 200 permutation tests (Figure 5C). The Q^2^ intercept values of −0.869 confirmed that the model was reliable and not overfitted.

To identify key metabolites responsible for quality differences between the varieties, a VIP plot was constructed. A total of 75 pivotal key metabolites were identified based on *p* < 0.05 and VIP > 1.0, utilizing tea metabolomics databases and relevant literature. These metabolites included 4 alkaloids, 10 phenolic acids, 19 amino acids and derivatives, 8 catechins and dimers, 24 flavonols and flavonol/flavone glycosides, 5 nucleotides and derivatives, and 5 organic acids. Detailed information regarding the m/z, RT, *p*-value, and VIP values for these metabolites is provided in Table 1. To assess their interdependence over varying spreading times, a heatmap was created, where the blue segment represents lower concentrations, and the red segment indicates higher levels (Figure 5D). These differential metabolites were then divided into four clusters based on hierarchical clustering analysis, reflecting their distribution across the SCGBT samples from the eight tea varieties. Cluster I primarily contained amino acids and derivatives, flavonol/flavone and flavonol/flavone glycosides, phenolic acids, and catechins. Within this cluster, amino acids and derivatives were predominantly higher in the HJY samples. Amino acids, key flavor components and aroma precursors, play an important role in the tea’s flavor profile (41, 47). On the other hand, compounds such as myricetin and (−)-epicatechin gallate, which contribute to the bitterness of black tea, were found in lower concentrations in HJY samples. Cluster II contained fewer compounds, such as N-acetylornithine, CAF, theaflavine-3,3-digallate, GA, epitheaflagallin 3-O-gallate, 5′-methylthioadenosine, (−)-gallocatechin, L-norleucine, and L-leucine. These metabolites exhibited the lowest levels in HJY, while they were more abundant in ZC302 and CC2 samples. Cluster III consisted of flavonol/flavone and flavonol/flavone glycosides, amino acids and derivatives, and organic acids. Representative metabolites included L-arginine, proline, rutin, kaempferol, kaempferol-7-O-glucoside, dihydromyricetin, dihydrokaempferol, kaempferol 3-O-sophoroside 7-O-rhamnoside, quercetin-3-O-rutinoside-7-O-rhamnoside, and fumaric acid. Flavonol/flavone and flavonol/flavone glycosides, known to contribute to the astringent taste of black tea (46, 48), were found in higher concentrations in FDDB, WNZ, and FX9 samples. Cluster IV included catechins, kaempferol and its glycosides, flavonol/flavone glycosides, amino acids and derivatives, phenolic acids, and organic acids. Overall, the heatmap analysis revealed that the most prominent metabolic variations between the different tea varieties were associated with catechins and dimers, amino acids and derivatives, flavonol/flavone and their glycosides, and phenolic acids.

**Table 1:** The detailed information of 75 differential metabolites among SCGBT samples processed by different varieties, including m/z, RT, fragments, *p* value, VIP value of PLS-DA model and identification grade.

No.	Compound	Mass error (ppm)	Detected *m/z*	RT/min	*p*-value	VIP
Alkaloids						
1	Caffeine	−1.4	194.1	3.1	<0.01	17.6589
2	Theobromine	−0.9	180.1	2.2	<0.01	8.33258
3	Acefylline	−3.9	190.1	1.3	<0.01	2.21058
4	Phenethylamine	0.0	121.1	2.4	<0.01	1.95689
Phenolic acids						
5	trans-Cinnamate	−1.7	148.1	1.7	<0.01	15.1287
6	4-p-Coumaroylquinic acid	−0.5	338.1	3.3	<0.01	3.68516
7	3,5-Dihydroxybenzoic acid	3.0	154.0	1.4	<0.01	3.45405
8	D-(−)-quinic acid	−0.4	192.1	0.7	<0.01	1.79731
9	Ellagic acid	−0.3	302.0	2.8	<0.01	1.69307
10	Shikimic acid	3.3	174.1	0.6	<0.01	1.60133
11	Gentisic acid	3.3	154.0	1.0	<0.01	1.49107
12	3-Aminosalicylic acid	3.2	153.0	5.7	<0.01	1.33602
13	Gallic acid	−0.5	170.0	1.3	<0.01	1.13344
14	p-Coumaric acid	−0.3	164.0	5.9	<0.01	1.07898
Amino acids and derivatives						
15	Isoleucine	−1.5	131.1	1.0	<0.01	12.2036
16	L-phenylalanine	−1.3	165.1	1.7	<0.01	12.3542
17	Homoproline	−0.5	129.1	0.7	<0.01	5.29181
18	Valine	−1.3	117.1	0.7	<0.01	4.38354
19	L-glutamic acid	−0.6	147.1	0.6	<0.01	3.20386
20	Proline	−0.1	115.1	1.4	<0.01	2.9112
21	L-tyrosine	−0.5	181.1	0.9	<0.01	2.73731
22	L-norleucine	−0.4	131.1	1.4	<0.01	2.66961
23	D-(+)-tryptophan	3.0	204.1	2.3	<0.01	2.45018
24	N-Acetyl-L-phenylalanine	3.2	207.1	1.0	<0.01	2.1881
25	L-leucine	3.1	131.1	1.0	<0.01	2.18472
26	L-arginine	−0.4	174.1	0.5	<0.01	2.152
27	Asparagine	−0.3	132.1	0.6	<0.01	2.12877
28	L-pyroglutamic acid	−0.6	129.0	0.6	<0.01	1.87194
29	N-acetylornithine	2.9	174.1	0.9	<0.01	1.6562
30	L-threonine	−0.6	119.1	0.6	<0.01	1.40901
31	L-alanine	−0.4	89.0	0.6	<0.01	1.36525
32	L-aspartic acid	−0.5	133.0	0.6	<0.01	1.23417
33	L-serine	−0.5	105.0	0.6	<0.01	1.20697
Catechins and dimers						
34	Catechin gallate	−0.4	442.1	3.8	<0.01	5.92968
35	(−)-Epicatechin	−0.5	290.1	3.3	<0.01	4.93239
36	Epigallocatechin gallate	−0.4	458.1	3.3	<0.01	2.98093
37	(−)-Epicatechin gallate	2.4	442.1	3.8	<0.01	2.50451
38	(−)-Gallocatechin	2.8	306.1	3.4	<0.05	1.64573
39	Epigallocatechin	3.6	306.1	2.2	<0.01	1.48043
40	Epitheaflagallin 3-O-gallate	−0.2	552.1	4.6	<0.01	1.44971
41	Theaflavine-3,3-digallate	−0.4	868.1	4.9	<0.01	1.006
Flavonol/flavone and flavonol/flavone glycosides						
42	Kaempferol-7-O-glucoside	−0.3	448.1	4.1	<0.01	4.59468
43	Kaempferol	−0.4	286.0	4.1	<0.01	5.13731
44	Kaempferol 3-O-sophoroside 7-O-rhamnoside	−0.6	756.2	3.7	<0.01	4.07382
45	Dihydromyricetin	−0.3	320.1	3.5	<0.01	4.0118
46	Dihydrokaempferol	2.9	288.1	4.6	<0.01	3.61564
47	Kaempferol-3-O-rutinoside	−0.3	594.2	4.0	<0.01	3.17144
48	Kaempferol-3-O-sophoroside-7-O-glucoside	−0.5	772.2	3.6	<0.01	2.69239
49	Astragalin	2.6	448.1	4.2	<0.01	2.64834
50	Quercetin	−0.3	302.0	3.7	<0.01	2.47118
51	Naringenin chalcone	−0.3	272.1	3.8	<0.01	2.27343
52	Rutin	−0.4	610.2	3.7	<0.01	2.27092
53	Isoquercetin	0.1	464.1	2.1	<0.01	2.26944
54	Myricetin	−0.3	318.0	3.5	<0.01	2.21952
55	Quercetin-3-O-rutinoside-7-O-rhamnoside	2.2	756.2	3.8	<0.01	2.07776
56	Kaempferol 3-glucorhamnoside	2.7	594.2	4.0	<0.01	2.04509
57	Luteolin	3.2	286.0	5.1	<0.01	2.00303
58	Kaempferol-3-O-(6′-galloyl)galactoside	0.1	600.1	4.1	<0.01	1.97036
59	Myricetin-3-O-galactoside	−0.2	480.1	3.5	<0.01	1.95402
60	Kaempferol 3- (2′-p-coumaryl-alpha- L-arabinopyranoside)	−0.4	564.1	4.7	<0.01	1.9517
61	Luteolin 7-xyloside	−0.1	418.1	4.3	<0.01	1.65074
62	Naringenin	−0.2	272.1	5.6	<0.01	1.64069
63	Kaempferol-3-O-(6′-trans-P-coumaroyl-2′-glucosyl)rhamnoside	0.0	740.2	5.0	<0.01	1.35947
64	Kaempferol-3-O-glucoside-7-O-rhamnoside	0.1	594.2	3.8	<0.01	1.15634
65	Quercetin-7-O-glucoside	−0.3	464.1	3.7	<0.01	1.08686
Nucleotides and derivatives						
66	Adenine	−1.1	135.1	0.7	<0.01	5.2265
67	Adenosine	−0.5	267.1	0.8	<0.01	2.98627
68	5′-Methylthioadenosine	−0.3	297.1	2.6	<0.01	2.95617
69	Guanine	2.1	151.0	0.8	<0.01	1.34447
70	7-Methylxanthine	−0.4	166.0	1.2	<0.01	1.26858
Organic acids						
71	Malic acid	2.9	134.0	0.6	<0.01	6.70401
72	Methylmalonic acid	2.9	118.0	0.7	<0.01	5.62366
73	5′-Glucosyloxyjasmanic acid	3.1	388.2	2.3	<0.01	3.02591
74	2-Isopropylmaleic acid	3.3	158.1	1.1	<0.01	2.06846
75	Fumaric acid	3.0	116.0	0.6	<0.01	1.72143

### 3.6 Correlation analysis of key differential metabolites and taste quality of sensory evaluation

**Figure 6:**
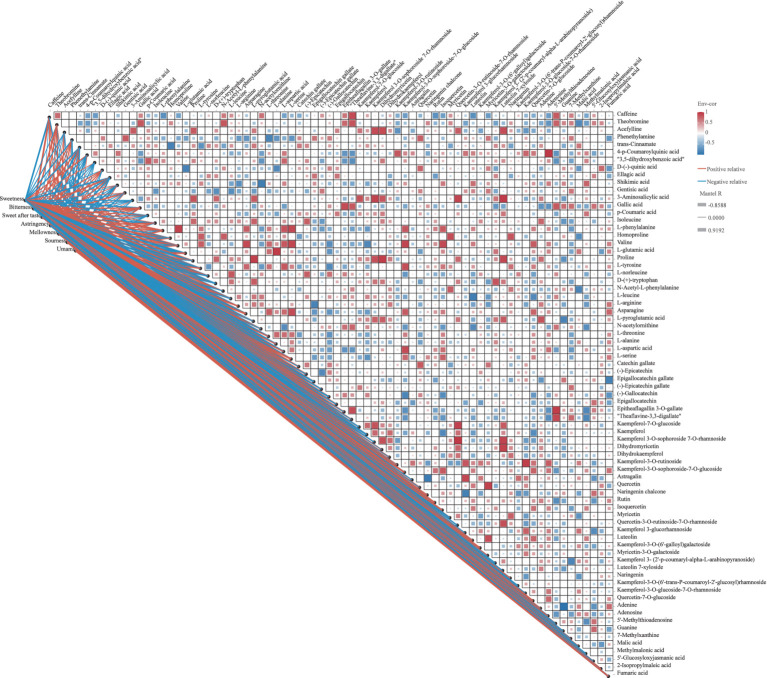
Correlation plot of key differential metabolites (*p* < 0.05, VIP > 1) and sensory indicators of SCGBT. In the Pearson correlation analysis, positive correlations are represented by red squares, and negative correlations by blue squares. In the Mantel test, red lines indicate positive correlations, while blue lines represent negative correlations; the line thickness correlates with the strength of the relationship.

The taste quality of tea results from the intricate interplay of various metabolite compounds. To explore the relationship between taste variations and phytochemical differences in SCGBT samples processed from different tea varieties, correlation analysis was conducted to assess the connection between the QDA taste attribute scores and key metabolites (VIP > 1.0, *p* < 0.05; Figure 6; Supplementary Table S2). Figure 6 highlights that blue indicates negative correlations, while red denotes positive correlations. Sweetness, a prominent sensory attribute of SCGBT, was strongly positively correlated with L-phenylalanine, valine, asparagine, L-serine, and kaempferol-3-O-sophoroside-7-O-glucoside (*r* > 0.7, *p* < 0.05), and negatively correlated with CAF, ellagic acid, GA, epigallocatechin gallate, (−)-epicatechin gallate, and kaempferol (|*r*| > 0.6). These metabolite content differences likely account for the variations in sweetness observed across SCGBT processed from different tea varieties. Bitterness exhibited a positive correlation with CAF, ellagic acid, L-norleucine, epigallocatechin gallate, (−)-gallocatechin, and rutin (*r* > 0.7), with the strongest correlation seen with epigallocatechin gallate (*r* > 0.7, *p* < 0.01). The substances negatively correlated with bitterness included L-tyrosine, asparagine, and fumaric acid (*r* > 0.7, *p* < 0.05). Additionally, (−)-epicatechin and epigallocatechin were positively correlated with the sweet aftertaste response (*r* > 0.6, *p* < 0.05). Previous studies have shown that the intensity of the sweet aftertaste increases with the molar concentrations of epigallocatechin and (−)-epicatechin, supporting these findings (41). The primary contributors to astringency included epigallocatechin gallate, (−)-gallocatechin, kaempferol, kaempferol-3-O-sophoroside-7-O-glucoside, rutin, and myricetin (*r* > 0.7). Flavones/flavonols and their glycosides significantly influence astringency due to their low sensory threshold (33, 42). Only L-norleucine, malic acid, and fumaric acid showed a positive correlation with sourness (*r* > 0.7). L-glutamic acid, asparagine, and L-serine exhibited significant positive correlations with umami (*r* > 0.8, *p* < 0.05), while umami negatively correlated with epigallocatechin gallate and (−)-gallocatechin. Interestingly, GA demonstrated a notable positive correlation with umami, aligning with previous findings that GA enhances the umami of green tea infusions and increases the umami strength of sodium L-glutamate (33, 49). No significant correlations were found between key metabolites and mellowness (*r* < 0.6, *p* > 0.05).

A deeper examination of the complex relationships among primary flavor components was conducted. As shown in Figure 6 and Supplementary Table S3, 51 metabolites with significant pairwise correlations (|*r*| > 0.6, *p* < 0.05) were identified. Regarding alkaloids, CAF exhibited a significant positive correlation with theobromine and theaflavine-3,3-digallate, and a negative correlation with L-phenylalanine. D-(−)-quinic acid demonstrated negative correlations with L-glutamic acid and L-alanine, while positively correlating with theobromine and theaflavine-3,3-digallate. Given quinic acid’s substantial effect on astringency and sourness, and the role of theobromine and theaflavine-3,3-digallate in bitterness, these metabolites likely influence the bitterness of green tea infusions (41, 42). In terms of amino acids and their derivatives, L-leucine, asparagine, and L-aspartic acid were key contributors to the umami and sweet flavors of tea. As depicted in Figure 6, L-leucine showed a strong positive correlation with L-norleucine, but a significant negative correlation with quercetin-7-O-glucoside (*p* < 0.05). Asparagine was highly positively correlated with valine (*p* < 0.01), and also positively correlated with L-threonine, L-aspartic acid, L-serine, kaempferol-3-O-sophoroside-7-O-glucoside, and quercetin-7-O-glucoside. L-aspartic acid exhibited significant positive correlations with L-phenylalanine, valine, and L-tyrosine. Epigallocatechin gallate negatively correlated with L-arginine, while (−)-gallocatechin negatively correlated with L-arginine, valine, L-glutamic acid, and L-tyrosine. In contrast, theaflavine-3,3-digallate showed positive correlations with epitheaflagallin 3-O-gallate and theobromine. Flavonols, flavones, and their glycosides displayed not only strong correlations among themselves, but also with amino acids, particularly valine and L-tyrosine, with correlation coefficients as high as 0.8 (*p* < 0.01). These results suggest that the flavor profile of SCGBT results from the synergistic or antagonistic effects of a wide range of compounds. Due to the complexity of black tea infusions, it is crucial to consider metabolite interactions in flavor development.

### 3.7 Impact of key metabolites on tea infusion taste

**Figure 7:**
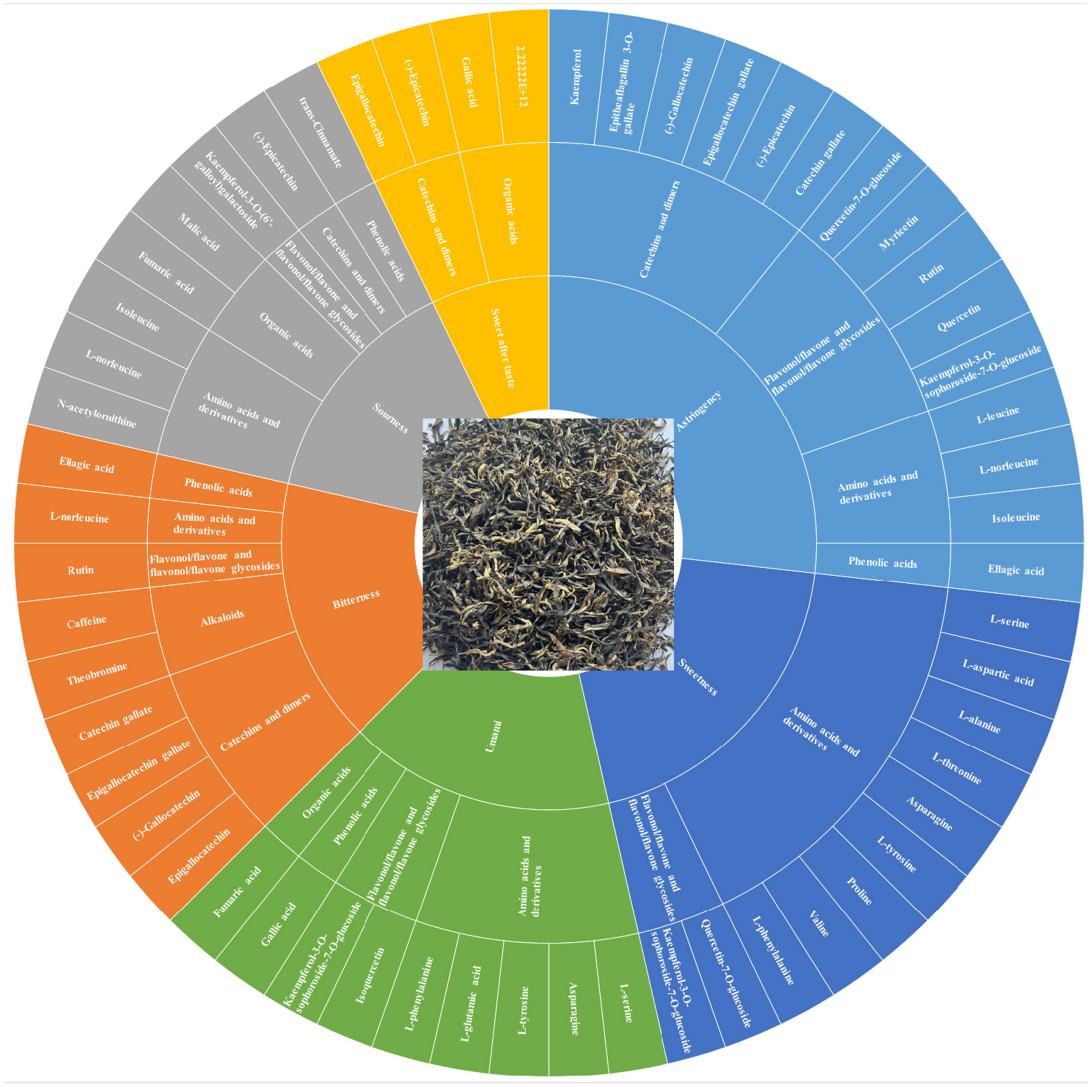
Flavor wheel highlighting the characteristic compounds associated with taste variations in SCGBT.

By integrating sensory evaluation with the analysis of key differential metabolites (VIP > 1, *p* < 0.05, *r* > 0.6), the link between flavor variations in SCGBT processed from different tea varieties and their associated chemical changes was further explored (Figure 7; Supplementary Table S2).

Umami and sourness: Sensory evaluation indicated that HJY had the highest umami, followed by MS131 and ZC302. Previous studies have identified FAAs as key contributors to the umami taste in tea infusions, with L-glutamic acid and asparagine recognized as primary contributors (41). In this study, L-glutamic acid and asparagine had the highest concentrations in HJY, with relative intensities of 1.20 × 10^9^ and 4.36 × 10^8^, respectively, followed by MS131 and ZC302. While the relative intensity of GA was highest (2.04 × 10^8^) in CC2, it is unlikely to be a direct contributor to umami, but rather an umami-enhancing compound due to its high taste threshold (49). Malic acid and fumaric acid, known to contribute to acidification during black tea storage (50), exhibited the highest relative intensities in HJY. However, sensory evaluation revealed that FX9 exhibited the highest sourness, potentially due to higher relative intensities of sweet and umami compounds in HJY compared to FX9.

Sweetness: The sweetness of black tea infusions is primarily attributed to carbohydrates and amino acids, such as glucose, sucrose, fructose, L-serine, L-alanine, proline, and L-threonine (41, 51). In this study, D-(+)-glucose and D-(−)-fructose were detected, but no significant differences in their relative intensities were observed across the SCGBT samples processed from eight varieties (*p* > 0.05). For sweet-tasting amino acids, the total relative intensities of proline, L-threonine, L-alanine, and L-serine were ranked as follows: WNZ > ZC302 > MS131 > HJY > FX9 > CC2 > FDDB > ZC108. However, the concentrations of these amino acids were low, and their dose-over-threshold factors were all below 0.1 (51). Therefore, further research is needed to better understand the contribution of these amino acids to the overall sweetness of black tea infusion.

Astringency: Astringency is a critical sensory attribute of tea, primarily influenced by hydrolysable and condensed tannins, which are considered detrimental to the sensory quality of black tea (41). Catechins, particularly ester catechins (EGCG and ECG), play a key role in the formation of astringency. In this study, EGCG exhibited lower relative intensities in ZC302, MS131, and HJY (Figure 5D), which is expected to significantly reduce the astringency in these three tea varieties. Furthermore, flavonol/flavone and their glycosides, which have much lower astringency thresholds compared to catechins, have been identified as essential compounds responsible for the velvety-like astringency in tea infusions (41, 43, 51). In this analysis, flavonol/flavone and their glycosides were present in lower relative intensities in HJY and ZC302, which may contribute to their reduced astringency. In contrast, higher relative intensities were observed in WNZ and ZC108.

Bitterness and sweet aftertaste: The lower bitterness perceived in HJY, MS131, and ZC302 contributed to the superior sensory quality of these samples compared to others. Catechins and CAF are known to be the primary contributors to bitterness in tea infusions (41, 42). In this study, four catechins were significantly altered (Figure 5D), with EGCG exhibiting the highest relative intensities, particularly in CC2 and ZC108, and lower intensities in HJY, MS131, and ZC302. Furthermore, the total amount of catechins was highest in ZC108 and lowest in HJY. The relative intensity of CAF was also lowest in HJY, suggesting that the reduction in both catechins and CAF contributes to the lower bitterness in HJY. Additionally, previous studies have suggested that rutin could enhance the bitterness of CAF (23). In this study, the variation in rutin components did not fully align with sensory evaluations, with higher relative intensities observed in HJY and MS131. In summary, despite the relatively higher levels of rutin, the significant decrease in EGCG and total catechins likely accounts for the lower bitterness in HJY, as indicated by sensory evaluation. The sweet aftertaste, which typically follows bitterness or astringency, is considered a positive characteristic in tea infusions (41). GA and non-gallated catechins, particularly (−)-epicatechin and epigallocatechin, are known contributors to the sweet aftertaste in green tea infusions (41). However, the relative intensity changes of these sweet aftertaste-related compounds did not align with sensory evaluations, further underscoring the complexity of the black tea flavor profile, which results from the combined effects of multiple compounds. These results suggest that ZC302, MS131, and HJY are more suitable for processing into higher-grade SCGBT based on their sensory and chemical profiles. Production experience demonstrates that processing coefficients influence how the tea quality develops (3, 8). In another study, researchers investigated volatile metabolites and non-volatile metabolites are not only transformed into each other and subject to synergistic or antagonistic effects by a variety of compounds (37). In the future, more efforts are needed to confirm the specific effects of these substances on tea taste quality.

## 4 Conclusion

This study provides a comprehensive and objective analysis of the relationship between taste and chemical components in SCGBT processed from different tea varieties, integrating sensory evaluation, quantitative analysis of major chemical constituents, and metabolomics. Sensory evaluation revealed that HJY, ZC302, and MS131 exhibited high intensities of sweetness, umami, and mellowness, with low bitterness and astringency. Compared to the other five tea varieties, these three had elevated levels of key taste-active compounds related to umami and sweetness, such as theanine, glutamic acid, aspartic acid, glycine, threonine, and serine. In contrast, CC2, FX9, and ZC108 had higher levels of ester catechins when processed into SCGBT, contributing to stronger bitterness and astringency. Furthermore, compared to HJY, ZC302, and MS131, the tea infusions of CC2, FX9, and ZC108 contained more compounds contributing to bitterness and astringency, likely explaining their more pronounced bitter and astringent tastes. A total of 75 key differential metabolites were identified (VIP > 1, *p* < 0.05), including 4 alkaloids, 10 phenolic acids, 19 amino acids and derivatives, 8 catechins and dimers, 24 flavonols and flavonol/flavone glycosides, 5 nucleotides and derivatives, and 5 organic acids. Correlation analysis revealed that metabolites such as CAF, GA, valine, L-glutamic acid, asparagine, L-serine, epigallocatechin gallate, kaempferol-3-O-sophoroside-7-O-glucoside, myricetin, and quercetin-7-O-glucoside had significant effects on SCGBT tea quality across different tea varieties. The higher relative intensities of amino acids (L-glutamic acid and asparagine) and phenolic acids (GA) were strongly associated with increased umami. The bitterness and astringency of the tea infusions were mainly influenced by the relative intensities of flavonol/flavone and flavonol/flavone glycosides, along with ester catechins (EGCG and CG). Sweetness was primarily driven by changes in the relative intensities of sweet-tasting amino acids (proline, L-threonine, L-alanine, and L-serine), while variations in non-ester catechins (EC and EGC) mainly accounted for changes in sweet aftertaste. In conclusion, this study underscores the importance of exploring the effects of different tea varieties on the quality of SCGBT. ZC302, MS131, and HJY are more suitable for processing into higher-grade SCGBT, and used the processing conditions was 24–26°C, relative humidity of 70–75% withering for 22 h, light pressure–heavy pressure–light pressure method rolling for 1.5 h, 22–25°C, moisture content ≥ 95% fermentation for 4.5 h, and first drying at 110°C and second drying at 80°C was helpful to improve the overall quality of SCGBT. These findings provide both a theoretical foundation and practical guidance for selecting high-quality raw materials for SCGBT production, which is crucial for the manufacturing of premium SCGBT tea.

## Data Availability

The original contributions presented in the study are included in the article/Supplementary material, further inquiries can be directed to the corresponding authors.

## Footnotes

^1^ https://app.rawgraphs.io/

^2^ https://www.omicshare.com/tools/

## Supplementary material

SUPPLEMENTARY FIGURE S1 Correlation plot among samples.

SUPPLEMENTARY TABLE S1 Sensory evaluation of eight SCGBT varieties.

SUPPLEMENTARY TABLE S2 Correlation analysis of key differential metabolites and sensory evaluation of taste quality.

SUPPLEMENTARY TABLE S3 Intricate relationship between primary flavor components.

## References

[1] Wang C Zhang CX Kong YW Peng XP Li CW Liu SH. A comparative study of volatile components in Dianhong teas from fresh leaves of four tea cultivars by using chromatography-mass spectrometry, multivariate data analysis, and descriptive sensory analysis. Food Res Int. (2017) 100:267–75. doi: 10.1016/j.foodres.2017.07.013, PMID: 28873687

[2] Jiang B Yang L Luo X Huang R Jiao W Zhong X. Aroma formation and dynamic changes during Sichuan black tea processing by GC–MS-based metabolomics. Fermentation. (2023) 9:686. doi: 10.3390/fermentation9070686

[3] Huang W Fang S Wang J Zhuo C Luo Y Yu Y. Sensomics analysis of the effect of the withering method on the aroma components of Keemun black tea. Food Chem. (2022) 395:133549. doi: 10.1016/j.foodchem.2022.133549, PMID: 35777211

[4] Schuh C Schieberle P. Characterization of the key aroma compounds in the beverage prepared from Darjeeling black tea: quantitative differences between tea leaves and infusion. J Agric Food Chem. (2006) 54:916–24. doi: 10.1021/jf052495n, PMID: 16448203

[5] Yang Z Baldermann S Watanabe N. Recent studies of the volatile compounds in tea. Food Res Int. (2013) 53:585–99. doi: 10.1016/j.foodres.2013.02.011

[6] Ma L Gao M Zhang L Qiao Y Li J Du L. Characterization of the key aroma-active compounds in high-grade Dianhong tea using GC-MS and GC-O combined with sensory-directed flavor analysis. Food Chem. (2022) 378:132058. doi: 10.1016/j.foodchem.2022.132058 35032805

[7] Qiu X Wang J Yu X Lv S Wu Y Wang C. Aroma formation in Dianhong black tea: effects of baking. Int J Food Prop. (2017) 20:2724–35. doi: 10.1080/10942912.2016.1249797

[8] Xu Y Liu Y Yang J Wang H Zhou H Lei P. Manufacturing process differences give Keemun black teas their distinctive aromas. Food Chem. (2023) 19:100865. doi: 10.1016/j.fochx.2023.100865, PMID: 37780253 PMC10534231

[9] Zhang Y Zhang P Le M Qi Y Yang Z Hu F. Improving flavor of summer Keemun black tea by solid-state fermentation using *Cordyceps militaris* revealed by LC/MS-based metabolomics and GC/MS analysis. Food Chem. (2023) 407:135172. doi: 10.1016/j.foodchem.2022.135172, PMID: 36508871

[10] Yao H Su H Ma J Zheng J He W Wu C. Widely targeted volatileomics analysis reveals the typical aroma formation of Xinyang black tea during fermentation. Food Res Int. (2023) 164:112387. doi: 10.1016/j.foodres.2022.112387, PMID: 36737972

[11] Kang S Yan H Zhu Y Liu X Lv H Zhang Y. Identification and quantification of key odorants in the world’s four most famous black teas. Food Res Int. (2019) 121:73–83. doi: 10.1016/j.foodres.2019.03.009, PMID: 31108802

[12] Kumazawa K Wada Y Masuda H. Characterization of epoxydecenal isomers as potent odorants in black tea (dimbula) infusion. J Agric Food Chem. (2006) 54:4795–801. doi: 10.1021/jf0603127, PMID: 16787030

[13] Jiang B Luo X Yan J Liu K Wang C Jiao W. Investigation of the effect of fragrance-enhancing temperature on the taste and aroma of black tea from the cultivar *Camellia sinensis* (l.) O. Kuntze cv Huangjinya using metabolomics and sensory histology techniques. Fermentation. (2024) 10:520. doi: 10.3390/fermentation10100520, PMID: 40225413

[14] Liu F Wang J Huang F Ye XL Tang XB Zhang T. Quality analysis and flavor wheel establishment of Sichuan congou black tea. Southwest China J Agric Sci. (2021) 34:1001–7. doi: 10.16213/j.cnki.scjas.2021.5.014

[15] Wang TT Luo XP Li LX Zhon XX Jiang B Yang LR. Effect of different drying technology on flavor and aroma of black tea. Food Re Develo. (2022) 43:121–8. doi: 10.12161/j.issn.1005-6521.2022.22.017

[16] Jin L Lian X Chen L Lei Y Li J Yang Z. Characteristic aroma analysis and interaction study of key aroma compounds of Chuanhong congou black tea. Eur Food Res Technol. (2024) 250:441–54. doi: 10.1007/s00217-023-04398-4

[17] Feng WZ Zhou H Xiong ZC Sheng CY Xia DZ Zhang JX. Exploring the effect of different tea varieties on the quality of Lu’an Guapian tea based on metabolomics and molecular sensory. Food Chem. (2024) 23:101534. doi: 10.1016/j.fochx.2024.101534, PMID: 38911473 PMC11192980

[18] Yue C Cao H Zhang S Hao Z Wu Z Luo L. Aroma characteristics of Wuyi rock tea prepared from 16 different tea plant varieties. Food Chem. (2023) 17:100586. doi: 10.1016/j.fochx.2023.100586, PMID: 36845464 PMC9945420

[19] Huang DJ Chen X Tang RR Wang HJ Jiao L Tang HY. A comprehensive metabolomics analysis of volatile and non-volatile compounds in matcha processed from different tea varieties. Food Chem. (2024) 21:101234. doi: 10.1016/j.fochx.2024.101234, PMID: 38420509 PMC10900760

[20] Bahorun T Luximon-Ramma A Gunness TK Sookar D Bhoyroo S Jugessur R. Black tea reduces uric acid and c-reactive protein levels in humans susceptible to cardiovascular diseases. Toxicology. (2010) 278:68–74. doi: 10.1016/j.tox.2009.11.024, PMID: 19963031

[21] Zhang H Qi RH Min Y. The impact of oolong and black tea polyphenols on human health. Food Bio. (2019) 29:55–61. doi: 10.1016/j.fbio.2019.03.009, PMID: 40276772

[22] Zhao T Huang X Zhao J Yang CS Zhang S Huang J. Theaflavins: an underexploited functional compound in black tea. Trends Food Sci Technol. (2024) 154:104755. doi: 10.1016/j.tifs.2024.104755

[23] Shan X Yu Q Chen L Zhang S Zhu J Jiang Y. Analyzing the influence of withering degree on the dynamic changes in non-volatile metabolites and sensory quality of Longjing green tea by non-targeted metabolomics. Front Nutr. (2023) 10:1104926. doi: 10.3389/fnut.2023.1104926, PMID: 36998915 PMC10043258

[24] Liu ZY Ran QS Luo JL Shen Q Zhang T Fang SM. Correlation analysis of secondary metabolites and disease resistance activity of different varieties of congou black tea based on LC-MS/MS and TCMSP. Food Chem. (2024) 23:101331. doi: 10.1016/j.fochx.2024.101331, PMID: 39071939 PMC11282962

[25] Gao J Chen D Xie D Peng J Hu Z Lin Z. Investigations of the highly efficient processing technique, chemical constituents, and anti-inflammatory effect of N-ethyl-2-pyrrolidinone-substituted flavan-3-ol (EPSF)-enriched white tea. Food Chem. (2024) 450:139328. doi: 10.1016/j.foodchem.2024.139328, PMID: 38626712

[26] Xiao LD Zhang LP Li SY Xu YY Wang W Liu N. Introduction performance of ten tea cultivars in Yibin area. China Tea. (2024) 46:44–51. doi: 10.3969/j.issn.1000-3150.2024.04.008

[27] Xia H Chen W Hu D Miao A Qiao X Qiu G. Rapid discrimination of quality grade of black tea based on near-infrared spectroscopy (NIRS), electronic nose (E-nose) and data fusion. Food Chem. (2024) 440:138242. doi: 10.1016/j.foodchem.2023.138242, PMID: 38154280

[28] Hua J Ouyang W Zhu X Wang J Yu Y Chen M. Objective quantification technique and widely targeted metabolomic reveal the effect of drying temperature on sensory attributes and related non-volatile metabolites of black tea. Food Chem. (2024) 439:138154. doi: 10.1016/j.foodchem.2023.138154, PMID: 38071844

[29] Wang Q Peng C Gong J. Effects of enzymatic action on the formation of theabrownin during solid state fermentation of Pu-erh tea. J Sci Food Agric. (2011) 91:2412–8. doi: 10.1002/jsfa.4480, PMID: 21656777

[30] Jiao YJ Cai M Zhang X Feng Z Zhang QZ Li LL. Impact of spreading time on flavor quality in Duyun maojian summer green tea. LWT. (2024) 214:117103. doi: 10.1016/j.lwt.2024.117103

[31] Qiao D Zhu J Mi X Xie H Shu M Chen M. Effects of withering time of fresh leaves on the formation of flavor quality of Taiping Houkui tea. LWT. (2023) 182:114833. doi: 10.1016/j.lwt.2023.114833

[32] Zhao M Ma Y Dai LL Zhang DL Li JH Yuan WX. A high-performance liquid chromatographic chromatographic method for simultaneous determination of 21 free amino acids in tea. Food Anal Methods. (2013) 6:69–75. doi: 10.1007/s12161-012-9408-4

[33] Ouyang J Jiang RG Xu H Wen S Liu CW Liu Y. Insights into the flavor profiles of different grades of Huangpu black tea using sensory histology techniques and metabolomics. Food Chem. (2024) 23:101600. doi: 10.1016/j.fochx.2024.101600, PMID: 39071923 PMC11283085

[34] Li Q Jin Y Jiang R Xu Y Zhang Y Luo Y. Dynamic changes in the metabolite profile and taste characteristics of fu brick tea during the manufacturing process. Food Chem. (2021) 344:128576. doi: 10.1016/j.foodchem.2020.128576, PMID: 33223295

[35] Li C Hu S Yang W Yang H Zhang W Ye J. Conversion obstacle from Mg-protoporphyrin ix to protochlorophyllide might be responsible for chlorophyll-deficient phenotype of the Huangjinya's albino offspring. Plant Physiol Biochem. (2024) 212:108778. doi: 10.1016/j.plaphy.2024.108778, PMID: 38838570

[36] Li N Yao Z Ning J Sun L Lin Q Zhu X. Comparison of different drying technologies for green tea: changes in color, non-volatile and volatile compounds. Food Chem X. (2024) 24:101935. doi: 10.1016/j.fochx.2024.101935, PMID: 39553236 PMC11564038

[37] Tian D Ma CY Zhou XH Yang LB Chen N Wang QY. Reveal the influence mechanism of different storage containers on the flavor of ripe Pu-erh tea based molecular sensory science. LWT. (2024) 214:117073. doi: 10.1016/j.lwt.2024.117073

[38] Liu XY Zhou F Wen MC Jiang S Long PP Ke JP. LC-MS and GC–MS based metabolomics analysis revealed the impact of tea trichomes on the chemical and flavor characteristics of white tea. Food Res Int. (2024) 191:114740. doi: 10.1016/j.foodres.2024.114740 39059930

[39] Long P Su S Wen M Liu X Han Z Ke J. An insight into trichomes-deficiency and trichomes-rich black teas by comparative metabolomics: the impact of oxidized trichomes on metabolic profiles and infusion color. Food Res Int. (2024) 190:114638. doi: 10.1016/j.foodres.2024.114638, PMID: 38945627

[40] Zhang L Ho CT Zhou J Santos JS Armstrong L Granato D. Chemistry and biological activities of processed *Camellia sinensis* teas: a comprehensive review. Compr Rev Food Sci Food Saf. (2019) 18:1474–95. doi: 10.1111/1541-4337.12479, PMID: 33336903

[41] Zhang L Cao Q Granato D Xu Y Ho C. Association between chemistry and taste of tea: a review. Trends Food Sci Technol. (2020) 101:139–49. doi: 10.1016/j.tifs.2020.05.015

[42] Shan X Deng Y Niu L Chen L Zhang S Jiang Y. The influence of fixation temperature on Longjing tea taste profile and the underlying non-volatile metabolites changes unraveled by combined analyses of metabolomics and E-tongue. LWT. (2024) 191:115560. doi: 10.1016/j.lwt.2023.115560

[43] Ye J Ye Y Yin J Jin J Liang Y Liu R. Bitterness and astringency of tea leaves and products: formation mechanism and reducing strategies. Trends Food Sci Technol. (2022) 123:130–43. doi: 10.1016/j.tifs.2022.02.031

[44] Feng J Zhuang J Chen Q Lin H Chu Q Chen P. The effect of maturity of tea leaves and processing methods on the formation of milky flavor in white tea - a metabolomic study. Food Chem. (2024) 447:139080. doi: 10.1016/j.foodchem.2024.139080, PMID: 38520904

[45] Wu Z Liao W Zhao H Qiu Z Zheng P Liu Y. Differences in the quality components of Wuyi rock tea and Huizhou rock tea. Food Secur. (2025) 14:4. doi: 10.3390/foods14010004, PMID: 39796294 PMC11720515

[46] Wang LL Xie JL Miao YW Wang W Hu JJ Jiang YW. Exploration of the effects of geographical regions on the volatile and non-volatile metabolites of black tea utilizing multiple intelligent sensory technologies and untargeted metabolomics analysis. Food Chem. (2024) 23:101634. doi: 10.1016/j.fochx.2024.101634, PMID: 39831178 PMC11740800

[47] Hu J Xie J Wang Q Tang J Zhou X Yuan H. Unraveling the dynamic variations of volatile and non-volatile metabolites in green tea during the yellow-light irradiation spreading process by targeted and untargeted metabolomics. LWT. (2025) 215:117152. doi: 10.1016/j.lwt.2024.117152

[48] Ito A Yanase E. Study into the chemical changes of tea leaf polyphenols during Japanese black tea processing. Food Res Int. (2022) 160:111731. doi: 10.1016/j.foodres.2022.111731, PMID: 36076419

[49] Kaneko S Kumazawa K Masuda H Henze A Hofmann T. Molecular and sensory studies on the umami taste of Japanese green tea. J Agric Food Chem. (2006) 54:2688–94. doi: 10.1021/jf0525232, PMID: 16569062

[50] Xie ZX Zhang D Zhu JY Luo QQ Liu J Zhou JT. Mechanism of aroma enhancement methods in accelerating congou black tea acidification subjected to room temperature storage. Food Chem. (2023) 438:137837. doi: 10.1016/j.foodchem.2023.137837, PMID: 37979270

[51] Scharbert S Hofmann T. Molecular definition of black tea taste by means of quantitative studies, taste reconstitution, and omission experiments. J Agric Food Chem. (2005) 53:5377–84. doi: 10.1021/jf050294d, PMID: 15969522

