# Supplementary figures and images for: Exploring the effect of different tea varieties on the quality of Sichuan Congou black tea based on metabolomic analysis and sensory science
Source: Front Nutr. 2025 May 9;12:1587413. doi: 10.3389/fnut.2025.1587413 (PMC12100625; doi:10.3389/fnut.2025.1587413)

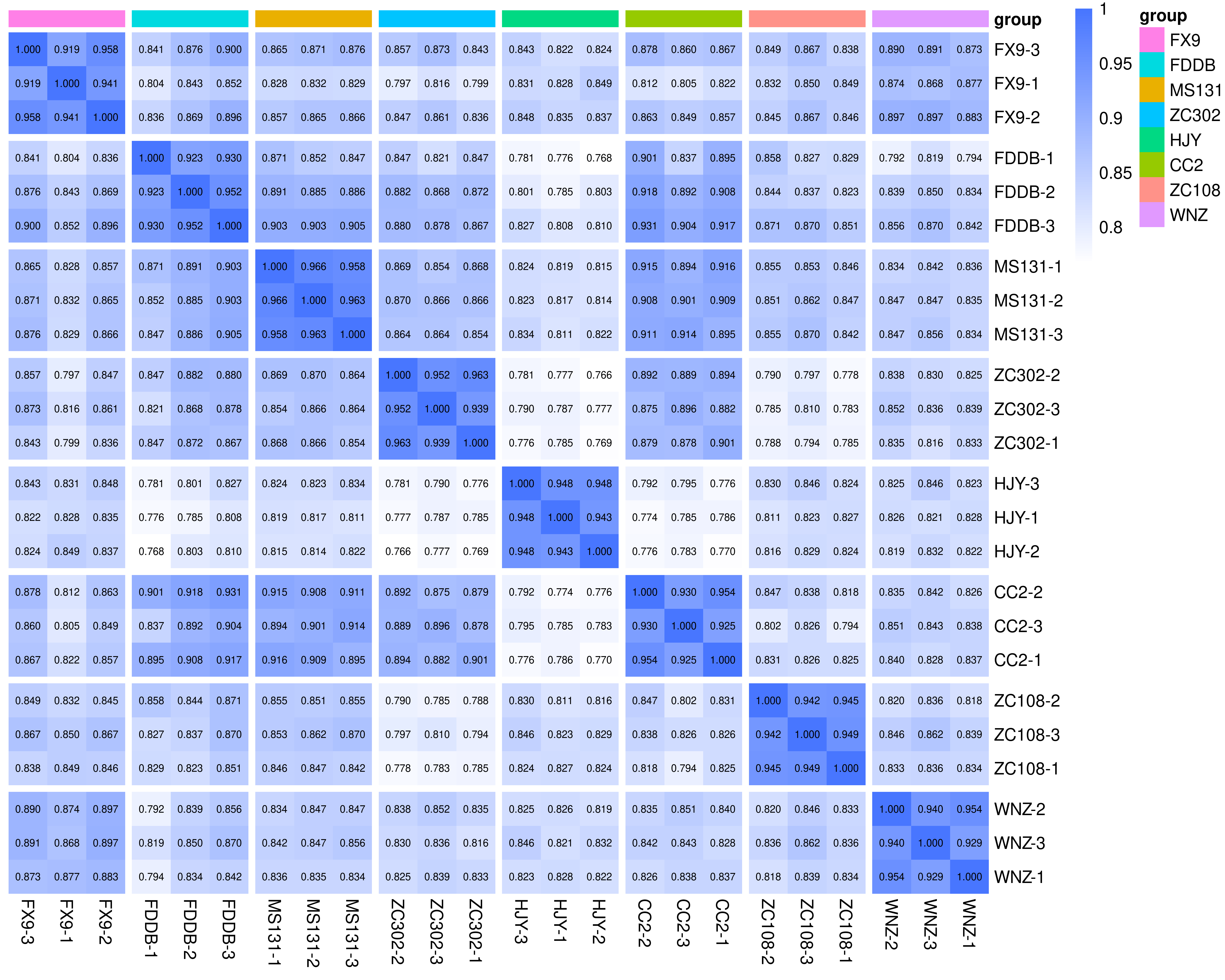

Supplement: SUPPLEMENTARY FIGURE S1 — Correlation plot among samples. [file Image_1.png]
