# Supplementary material for: Exploring the effect of different tea varieties on the quality of Sichuan Congou black tea based on metabolomic analysis and sensory science
Source: Front Nutr. 2025 May 9;12:1587413. doi: 10.3389/fnut.2025.1587413 (PMC12100625; doi:10.3389/fnut.2025.1587413)
Supplement: SUPPLEMENTARY TABLE S1 — Sensory evaluation of eight SCGBT varieties. [file Table_1.doc]

Table S1. Sensory evaluation in eight varieties of SCGT

| **Samples** | **Appearance (25%)** | | **Liquor color (10%)** | | **Aroma (25%)** | | **Taste (30%)** | | **Infused leaf (10%)** | | **Total quality score** |
| --- | --- | --- | --- | --- | --- | --- | --- | --- | --- | --- | --- |
|  | **Comment** | **Score** | **Comment** | **Score** | **Comment** | **Score** | **Comment** | **Score** | **Comment** | **Score** |  |
| ZC302 | Curly and tight, black bloom with show golden pekoe | 91.0±0.51 | Red, brighter | 93.0±0.71 | Sweet and fruity aroma | 92.0±1.20 | Mellow and thick taste, sweet after taste | 91.5±0.80 | Tender, even red and bright | 90.5±0.85 | 91.7±0.74 |
| FDDB | Curly and tight, black bloom with more golden pekoe | 89.0±0.76 | Red, brighter | 90.5±0.83 | Sweet and pure | 90.5±0.95 | Sweet, slightly sourness | 87.0±0.85 | Tender, even red and bright | 90.0±1.10 | 89.0±0.85 |
| WNZ | Curly and tight, black bloom with have golden pekoe | 86.0±0.65 | Red, brighter | 91.0±0.80 | Sweet and pure | 88.5±1.31 | Sweet and mellow | 90.5±1.20 | Tender, even red and bright | 89.0±1.14 | 88.8±0.92 |
| CC2 | Curly and tight, black bloom with Show golden pekoe | 91.8±0.45 | Red, brighter | 90.0±0.64 | Sweet with slightly  grassy | 85.5±0.93 | Sweet, slightly astringent | 87.5±0.65 | Tender, even red and bright | 90.0±0.90 | 88.6±0.50 |
| FX9 | Curly and tight, black bloom with more golden pekoe | 88.7±0.43 | Red, brighter | 90.6±0.51 | Sweet and pure | 88.5±1.31 | Sweet and mellow | 90.0±0.84 | Tender, even red and bright | 90.0±1.16 | 89.4±0.70 |
| MS131 | Curly and tight, black bloom with show golden pekoe | 90.6±0.65 | Red, brighter | 93.0±0.76 | Sweet with floral  aroma | 91.5±1.35 | Mellow and thick taste, sweet after taste | 91.5±1.10 | Tender, even red and bright | 90.5±0.94 | 91.3±0.65 |
| ZC108 | Curly and tight, black bloom with have golden pekoe | 87.1±0.61 | Red, brighter | 90.5±1.10 | Sweet with slightly  grassy | 87.8±1.32 | Sweet and mellow | 90.5±0.93 | Tender, even red and bright | 90.0±1.14 | 88.9±0.85 |
| HJY | Curly and tight, black bloom with have golden pekoe | 86.8±0.81 | Orange-red, brighter | 87.0±0.86 | Sweet and fruity aroma | 92.5±1.23 | Mellow and thick taste, sweet after taste | 92.5±1.50 | Tender, even red and bright | 90.5±0.95 | 90.3±1.10 |
