# Supplementary material for: Exploring the effect of different tea varieties on the quality of Sichuan Congou black tea based on metabolomic analysis and sensory science
Source: Front Nutr. 2025 May 9;12:1587413. doi: 10.3389/fnut.2025.1587413 (PMC12100625; doi:10.3389/fnut.2025.1587413)
Supplement: Supplementary file 5 [file Supplementary_file_1.doc]

**Non-targeted metabolomics analysis**

The sample extraction method was modified based on previous methods(33, 34). Briefly, 0.05 g of tea powder and 1 mL of an acetonitrile–methanol–water (45:45:10, v/v/v) solution containing N-acetyl-D-alloisoleucine (20 mg/L) as an internal standard were added to a 5 mL centrifuge tube. The mixture was then subjected to ultrasonication for 10 minutes and left to stand for one hour at –20°C. Next, the supernatant was centrifuged at 12,000 g for 15 minutes at 4°C. The supernatant was then aspirated and filtered for subsequent analysis. Three extraction replicates were performed for each sample. Quality control (QC) samples were obtained by mixing equal amounts from each tea sample.

For metabolomics analysis, a liquid chromatography-mass spectrometry (LC–MS) system consisting of an Ultim3000 ultra-high-performance liquid chromatography system coupled with an Orbitrap Exploris 480 high-resolution mass spectrometer was used. The column used for LC separation was a Waters Acquity UPLC HSS T3 column (1.8 µm × 2.1 mm × 100 mm) running at 35°C. The mobile phase consisted of 0.1% water–formic acid (A) and 0.1% acetonitrile–formic acid (B). The elution gradient was as follows: 0–0.25 min: 2% B; 0.25–10 min: 2–98% B; 10–13 min: 98% B; 13–13.1 min: 98–2% B; 13.1–15 min: 2% B. The flow rate was set to 300 μL/min, and the injection volume was 1 μL. The Q Orbitrap mass spectrometer was used for acquiring MS/MS spectra on a data-dependent basis during an LC-MS experiment. The scanning range was m/z 67–1000. The parameters of the electrospray ionization (ESI) ion source were as follows: spray voltage: 3500V (positive ion mode) and 2500V (negative ion mode); sheath gas flow rate: 50 arb; auxiliary gas flow rate: 10 arb; sweep gas flow rate: 1 arb; ion transfer tube temperature: 325°C; vaporizer temperature: 350°C. The raw data acquired by LC-MS were imported into the CD search library software for further analysis. Peak extraction, peak alignment, m/z, and retention time corrections were performed. Peak alignment was carried out on different samples with a retention time deviation of 0.2 minutes and a mass deviation of 5 ppm to improve identification accuracy. Peak extraction was conducted based on a mass deviation of 5 ppm, a signal intensity deviation of 30%, S/N ≥ 3, signal intensity ≥ 100,000, and additive ions. The peak area was quantified, and the target ions were integrated. Metabolic identification information was obtained by searching the laboratory’s mzCloud, MZVault, and ChemSpider databases, while theoretical fragments were integrated and quantified. QC samples were run every 10 samples to monitor repeatability throughout the analysis.
